# American Proctologic Society

**Published:** 1909-10

**Authors:** Lewis H. Adler

**Affiliations:** Philadelphia


### SOCIETY PROCEEDINGS.

### American Proctologic Society

REPORTED BY LEWIS H. ADLER, JR., M. D., Philadelphia, Secretary.

*Eleventh Annual Meeting, held at Atlantic City, N. J., June 7 and 8, 1909.*

### INTESTINAL AUTOINTOXICATION: ITS TREATMENT BY IRRIGATION, by William L. Dickinson, M.D., Saginaw.

DURING normal digestion, there are present in the intestine peptones, crystalline bodies, aromatic substances and ptomaines, which are toxic, but changed into less toxic bodies and eliminated by the stools. Whenever their number is very great, relief is obtained by a profuse intercurrent diarrhea, while the remaining toxic bodies, having been acted upon partially by the digestive mucosa, are changed in the liver, then enter the circulation, and being further changed by the antitoxic glands, finally are eliminated through the skin, kidneys and lungs.

Many patients have suffered for years, and perhaps the greater part of their lives from constipation, and the condition has been aggravated as they have grown older and more sedentary in their habits.

There are well-marked symptoms in the autointoxicated. Among the prominent are: a drawn expression; sunken eyes; frequently the so-called liver spots; often the patient is pot-bellied and the skin is dry and harsh; it is quite common to have the bowels greatly distended by gases, shortly after meals, neces-

5. Lancet, May 1, 1909, "A Clinical Lecture on the Removal of Tumors of the Large Intestine," by Arthur E. Barker, F.R.C.S.

sitating the loosening of the clothing; the breath is frequently very offensive; the odor of the stools is sickening, while the stools are constipated, hard, lumpy, and of small caliber or semiliquid and mushy, and upon examination mucus and membranes are found. Patients are often unable to concentrate their thoughts, and there is loss of memory. There is great fatigue, and depression of spirits. Pruritus, urticaria, eczema or furunculosis caused by intestinal autointoxication may be present.

These are not all the symptoms that may arise from intestinal autointoxication but they are sufficient to emphasize the importance of the subject, and the necessity of having the intestinal discharges examined by a competent person before and during the treatment of the patient. An examination of the urine to determine the amount of indican present in cases of intestinal autointoxication can be made by any physician, but there are times when a laboratory examination must be made by an expert.

The treatment must of necessity begin with careful attention to the kind and amount of food taken. Vegetables should largely replace meats, and in fact the patient will gain faster if meat is not partaken of at all. There should be a liberal use of water internally,—drinking between meals two to three quarts of water daily.

The treatment is not simple and is one that requires attention and generally a long time. The routine method is the administration of calomel gr. 1-10 and podophyllin gr. 1-24 repeated every hour for eight or ten doses, followed with rochelle salt one-half ounce in six ounces of hot water every two hours until the stools are watery. The colon should be distended with warm water containing half an ounce of soda sulphate to the quart. The patient should be in the knee-chest position. The water should flow slowly, fully distending the bowels, but not causing pain. This washing out of the bowel should be done daily for about one week and the urine should be examined again for indican, and if it is found present, the indication is that there is need of another course of the calomel and podophyllin. The bowel should be made aseptic by the use of sulphocarbolate of zinc gr. x to one quart of water used by enemata retaining as much of it as possible.

The treatment is to keep the intestine as clean as possible.

"Perirectal Abscess," by J. A. MacMillan, M.D., Detroit, Mich.

Who called attention to the fact that in a large proportion of cases of perirectal abscesses, the bacillus tuberculosis is present, and that next in importance, as an etiologic factor, is the gonococcus. A diagnosis is most difficult when the abscess is located above the levator ani. In this location it is frequently found to be complicated with some disease of one or more of the pelvic organs. In this condition it is sometimes necessary to make an abdominal incision both for exploratory purposes and to rectify the condition. In the treatment of the perirectal abscess, however, the drainage should always be from below.

Disease of the Colon due to Extraintestinal Causes, with Special Reference to Membranous Colitis: Illustrative Cases, by A. B. Cooke, M.D., Nashville, Tenn.

The intimate functional relations existing between the several viscera of digestions, which is recognised by all, was pointed out, but the writer stated that the anatomic relations of the alimentary tube and the frequency with which they are to be looked to for the explanation of many of its pathologic conditions, have not received the serious consideration their importance demands. He also called special attention to certain familiar diseases of the colon, which are often found to exist primarily because of these relations, and the mechanical irritation growing out of them.

Perhaps, the most conspicuous of which, was cited *membranous colitis*. The writer recalled the great divergence of opinion that has always prevailed as to the true nature and pathology of this malady, and notwithstanding the conclusions of such authorities as Osler, Tyson, Hemmeter and others, that the disease is a secretion neurosis; he takes the contrary view held by many other equally careful and competent clinicians, who hold that there are always pathological lesions that bear directly upon the colon, either from without, as by pressure from other misplaced organs, or by adhesions, or by some local irritant from within to account for these cases.

For present purposes the term membranous colitis is limited to that peculiar affection, which is characterised by the periodic discharge of mucus with membranes or casts from the bowel, and of which fecal stasis is always a prominent feature. With reference to this type of colitis, Dr. Cooke stated unequivocally that he had never seen a case in which he failed to find some gross pathologic condition of one, or more abdominal organs as well as of the mucosa itself; and furthermore that the etiologic relation between the two has been clearly established in a number of cases by the prompt and permanent disappearance of the bowel trouble upon correction of the extraintestinal condition, after all other methods of treatment had failed. From this experience he had been led to conclude that the primary causes of this particular variety of colitis belongs in the main, if not exclusively, to a special class, viz., those which act mechanically.

Most noteworthy in the list of such causes are enteroptosis, right movable kidney, peritoneal adhesions and extraintestinal growths which occasion continuous pressure upon some portion of the colon.

He then discussed each of these causes in detail and supported his argument by the enumeration of well-illustrated cases.

Necessity for Routine Examination of the Rectum in Intestinal Diseases: Illustrated Cases, by Dwight Henderson Murray, M.D., Syracuse, N. Y.

Dr. Murray's paper was one of special interest to the general practitioner and emphasized the necessity for rectal and colonic examinations in all cases of protracted diseases of the digestive tract, whether special symptoms are directed to the rectum and colon or not.

In many cases of gastrointestinal disturbances the real cause may be found in the rectum or colon, if sought, though the patient gives no symptoms of such rectal trouble. These are amenable to local treatment.

A thorough examination including rectal and bacteriological examination of the stools, should be made in every chronic intestinal case before beginning treatment. He advised that physicians should not treat patients who refuse to allow the necessary examination.

He reported illustrative cases including so-called intestinal indigestion and dyspepsia, chronic diarrhea, cancer of the sigmoid, and internal hemorrhoids.

A case of internal hemorrhoids where the attending physician had entirely neglected to examine the rectum, had been treated by lavage seven months, for so-called dyspepsia and dilation of the stomach without benefit, and was told that a gastroenterostomy was the only hope of cure. After an operation for radical removal of the internal hemorrhoids he was cured of his dyspepsia. A careful diagnosis would have saved this patient years of suffering.

The patient's life in one instance (possibly) and certainly the general reputation of the medical profession in all of the cases would have been better had the patients been carefully examined.

This neglect was found to be true not only of the physicians in this country, but of physicians in Europe, who had treated some of the cases in the list reported.

The author made a plea not only for local but bacteriological examination, claiming that every case of diarrhea, continuing for a longer time than is sufficient for nature to eliminate the irritating material that may be causing it, is due to a more serious disease.

There are many local conditions that cause a chronic diarrhea which would be eliminated by a simple operation or local treatment. When allowed to become chronic while depending upon oral medication, frequently the time when a cure could be affected, had passed, and chronic invalidism or death may result.

Sir Charles Ball's Operation for Internal Hemorrhoids, by G. W. Combs, M.D., Indianapolis, Ind.

In which he briefly described the operation advised by Mr. Ball for the removal of internal hemorrhoids which consists: (1) of making a curved incision opposite the pile being treated, terminating in the mucous membrane on either side of the pile, the greatest convexity not including more than one-third of the revoluted anal ring; (2) of bluntly dissecting the pile from the external sphincter, the dissection being carried upward until healthy mucous membrane is reached; (3) of crushing the pedicle in a powerful clamp; (4) of passing a heavy silk ligature subcutaneously in the remaining two-thirds of the revoluted anal ring and through the crushed mucous membrane pedicle, one part of which is constricted in a first tying and the whole of it in a second; (5) of tying the ligature very tightly, thus bringing the remaining two-thirds of the revoluted anal ring up into position, maintaining it there until union takes place and constricting the pedicle so that sloughing will occur.

The results obtained by the writer have not been so favorable as those that should follow the procedure as indicated by the author.

The following are the writer's conclusions:

1. The post-operative pain is greater than after the usual ligature or clamp and cautery method.

2. The duration of the healing period is not shortened because of the sloughing of the ligature from either the skin or pedicle before union takes place, leaving the wounds to heal by granulation.

3. There is a necessity for unusual watchfulness that all ligatures may be removed as they slough.

4. Failing to secure primary union, skin-tabs frequently remain for subsequent removal.

5. No time is saved by this modification of the ligature operation.

6. There is danger of secondary hemorrhage from an early tearing off of the pedicle by traction.

The Technic of the Injection Treatment for Hemorrhoids, by Edwin A. Hamilton, of Columbus, Ohio.

He stated that the injection treatment does not have a wide application; as its indiscriminate use is followed by embolus, abscess and other complications and relapses are prone to occur except in cases especially adapted to this method. The instruments needed are a cone-shaped anal speculum with one broad fenestrum and a special copper-tipped long needle of large caliber with an outside barrel which may be screwed to the needle proper to regulate the depth to which it may be inserted. The solution is 10 per cent. carbolic acid, 90 per cent. oil of sweet almonds. Neither water nor glycerine is used in the solution as they cause pain. When the sphincter is normal or hypertrophied, the hemorrhoids are never strained outside of the rectum and treated there, but are allowed to protrude through the fenestrum of the speculum and attended to in their normal location. In cases where the sphincter is dilated and the hemorrhoids are easily replaced, they may be treated outside, but under no other conditions. From four to eight drops are injected in a hemorrhoid, only one injection being made at one treatment. The patient rests in the recumbent posture for several minutes. No application or dressing is applied. The bowels are moved after the second day. Subsequent treatments may be administered at intervals of five days.

The Test Diet; Nitrogen and Sulphate Partitions, as an aid to Diagnosis in Intestinal Disturbances, by Jerome M. Lynch, M.D., New York City, N. Y.

Who stated that the subject of test-diet, as suggested by Professor Schmidt, is one well worthy of study. If, after a proctoscopic examination of the rectum and sigmond,—and an examination of the stomach contents, a case is still obscure, the test-diet should be given, and an examination of the feces and a thorough examination of the urine, with nitrogen and sulphate partitions, be made. Otherwise, one cannot conscientiously say he has exhausted all the resources at his command.

These tests, he admitted, are not always conclusive, but in most cases they are of great help; often, a positive solution of doubtful problems.

Of twenty-five cases under observation during the last six months, he found three of especial interest. Case I, was referred for treatment on account of moderate diarrhea, with prolapsing and bleeding internal hemorrhoids. The stomach had been previously examined with negative results. Proctoscopic examination, except for hemorrhoidal condition was negative. Put on test-diet. The specimen of feces examined had a somewhat pasty consistency, a light yellow color, normal odor and showed no macroscopic admixture. Microscopic examination showed the usual amount of striped muscle fiber, carbohydrate food remnants and granular detritus, with an excess of free fat and fatty acids. The starch was properly digested; bacterial flora, not excessive; reaction, neutral. Sublimate test, negative. Fermentation test, negative. The specimen showed evidence of deficient bile admixture.

The analysis of a twenty-four hour specimen of urine showed the specimen to contain no albumen and no renal elements, with a normal daily amount of urine, a normal specific gravity and a normal daily excretion of urea. The sulphate ratio as well as the ratio of the urea and uric acid was somewhat depressed, with the presence of a marked excess of indican.

Analysis of this report disclosed at once the cause of the diarrhea, namely: deficiency of bile with excess of fatty fluids and depressing of sulphate ratio, causing autointoxication.

The other two cases were equally interesting.

Relative to the determination of the clinical significance of faulty sulphate and nitrogen partition, the writer stated that the relative increase in ethereal sulphate may be due to one of several causes, among which were mentioned, stasis in the bowel, ingestion of decomposing nitrogenous food, improper digestion of food in the stomach and upper intestine, by diminution or absence of hydrochloric acid and bile, the result of excessive or faulty bacterial fermentation in the lower portion of the small intestine and the upper portion of the large intestine. This process may exist without an actual toxemia, and an actual toxemia may exist without this particular putrefactive process; but they are usually associated.

Excess of ethereal sulphate is usually associated with an excess of endoxyl sulphate, though not always. Without means of estimating the amount of the actual products of toxemia, the relative excess in ethereal sulphates is used as a guide, although subject to errors, as are other guides.

Fault in the nitrogen partition would seem to justify the inference that the hepatic function is disturbed. The decrease in the relative amount of urea nitrogen probably indicated the degree of the fault. With this decrease, there is a relative increase in the amount of one or more of the other forms of nitrogen in the urine. In the severe toxemias of pregnancy, pneumonia, etc., this is chiefly in ammonia nitrogen and creatinin nitrogen; in digestive disturbances the increase in the so-called extractive nitrogen, and in lithemic cases and in those of cyclic vomiting, headache, or albuminuria, in the purin nitrogen as well, particularly during the acute attack. In cases of enteritis or colitis, owing to the destruction of cells, the purin nitrogen is often increased.

Faulty nitrogen partition may exist without a toxemia but a hepatotoxemia without a faulty nitrogen partition is practically unknown. Acidosis frequently accompanies a faulty nitrogen partition; but it would seem an evidence of the toxemia rather than of the fault in hepatic function, though this is disputed by some.

Multiple Adenomata of the Rectum, by James P. Tuttle, M.D., New York City.

Stated that the distinction between multiple adenomata and polypi is more marked clinically, than histologically. Pendunculated adenomata or polypi may exist in varying numbers without constituting a true multiple adenomata. Age and its relation to the two types; distinction between the two types in proportion to the number of growths; the relative frequency of the growths in different portions of the bowel; growths found largely in the sulci and not in the mucous folds of the bowel. What is the probability of malignant metamorphosis when not interfered with? The tendency to recurrence, in malignant form, after surgical measures? Results of internal and local medication; results of functional rest to the parts. Does radical operation furnish the best hope for the patient, in view of clinical experience?

Surgical Treatment of Diarrhea and a Description of a New Cecostomy which Permits free Irrigation of both the Small and Large Intestine, by Samuel Goodwin Gant, M.D., LL.D., New York City, N. Y.

In this article attention was first called to the frequency of occurrence of chronic diarrhea and the simplest and most reliable methods were briefly outlined of diagnosing ulcerative lesions of the colon inducing diarrhea and also the relative frequency was mentioned between gastric and hepatic diarrhea and those caused by local disease of the large intestine. The author then proceeded to make the following points:

1. That acute attacks of diarrhea could sometimes be controlled by diet, rest and internal medication and, further, that the frequency of the evacuations could occasionally be diminished by these therapeutic measures in chronic diarrhea but that a cure of the latter could be accomplished only in rare instances in this way.

2. That the treatment of chronic ulcerative colitis by internal medication should be abandoned because it is harmful in many ways and utterly unreliable in so far as a cure of the diarrhea is concerned.

3. That *direct bowel treatment* by lavage or medicated irrigation introduced through the anus or from above through the appendix or cecum, is the only rational treatment for diarrhea due to ulcerative lesions of the colon.

4. That operative procedures are contradicted except in cases where, for any reason, the colon tube cannot be introduced sufficiently high, to insure thorough washing out of the entire large bowel and when operative procedures are declined.

5. That the surgical treatment of chronic diarrhea gives universal satisfaction and that he recommended appendicostomy and cecostomy for the relief of this ailment with the same confidence that he did appendectomy for appendicitis.

6. The relative values of *resection, intestinal exclusion,* colostomy, *appendicostomy, simple cecostomy,* and *cecostomy* with an arrangement for irrigating the small intestine (Gant's operation), in the treatment of chronic diarrhea, were fully discussed. The results of his experience show that appendicostomy and cecostomy could be performed most quickly, were the least dangerous, give the best results and were less often followed by unpleasant sequellæ than the other procedures.

7. He stated that formerly he was prejudiced in favor of appendicostomy but that a more recent and larger experience had caused him to look with greater favor upon cecostomy; especially when combined with irrigation of the small intestine. He maintained that his cecostomy was suitable in all cases of chronic diarrhea because it could be employed when the frequent stools were due to both an enteritis and an ulcerative colitis and when the lesions were confined to the colon alone, and, further, that his operation should supercede appendicostomy, in many instances, because the appendix was frequently unfit for irrigating purposes, because it was too short, too narrow, strictured or bound down by adhesions and often had a tendency to become necrotic, slip back into the abdomen, become closed when not kept open by the introduction of a catheter and that appendicostomy was not suitable when the small bowel was diseased.

8. He then briefly described the technic of his cecostomy with provision for small intestine irrigation, the main idea of which consisted in making an openinig in the cecum and inserting two tubes, one into the cecum and the other into the small intestine through the ileo-cecal valve by the aid of a catheter-carrier. He claimed that the advantage of this procedure over other operations, was that either the small or large bowel could be irrigated at will and that there was no fecal leakage about the catheters.

9. In concluding his remarks, he summarised the results obtained by him in the surgical treatment of chronic diarrhea by the through and through method and reported 38 cases treated by appendicostomy, and 14 by cecostomy, 8 of the latter being operated upon by the Gibson, and the remainder by his new procedure and said that the universally successful results obtained by surgery in this class of cases is far better than those obtained by the use of the time-worn way, where they depend upon dieting, rest and medication, as practised by many physicians today.

A Report of Two Cases of Anomalous Sigmoid, by Arthur Hebb, M.D., of Baltimore, Md.

One case was an extremely long sigmoid, reaching from the mammary line to a point midway of the thighs, when withdrawn from the abdomen; the second case was a short sigmoid, with a mesentery ¾ inches in length, situated above the crest of the ilium, on a line with the lower border of the last rib, coming off from the descending colon. It was only 4 inches in length. The descending loop, with no mesentery, ran down over the bifurcation of the left iliac artery and ureter; then forward, hugging the left side of the pelvis and down over the anterior and posterior branches of the internal iliac artery where it joined the rectum.

Nevus of the Anal Region with Report of a Case Associated with Internal Hemorrhoids, by Lewis H. Adler, Jr., M.D., Philadelphia, Pa.

The author of this paper mentioned the rarity of this condition as an anal affection. The patient whose condition was detailed was a male, aged 40, whose habits were good. From birth he had a noticeable fullness at the anus, which as he grew older occasioned him considerable annoyance when walking and at stool. When twenty years old he had had an operation for hemorrhoids performed, which temporarily gave relief. As time went on his hemorrhoidal trouble returned and the external congenital fulness became worse. Bleeding frequently attended efforts to have an evacuation, though the bowels were never, what might be called costive.

Examination prior to operation, revealed a mass of thickened skin, of a dull purplish hue, surrounding the anus, about two inches in width and elevated from the surrounding skin about 1|16 of an inch. Scattered over this area were numerous hairs. The anus was quite patulous, and, upon bearing down, a hemorrhoidal mass protruded and the external portion, around the anus, visibly increased.

A diagnosis was made of nevus associated with internal hemorrhoids, and an operation was advised to which the patient readily consented. At this time, he was apparently in fair physical condition and by no means markedly anemic, although his color was far from normal and he lacked what might be termed resistance. His weight at the time was 151 pounds and his usual weight being stated to have been 170 pounds.

An operation was performed on March 29th, five days after he was first seen by the writer. The patient took the anesthetic very badly; it requiring over a half hour to get him in a condition to be placed upon the operating table. After the removal of the hemorrhoids, which were as large as any the writer had ever seen, —the tissue composing them being much thicker and denser than is usually encountered in ordinary cases—the patient's condition was that of profound collapse. The usual clamp and cautery method was used for the removal of the five hemorrhoidal masses present. After the administration of a hypodermic injection of atropin and strychnine, the patient rallied, and the nevus was then excised. The removal of the latter caused very little loss of blood, so much so, that its absence was remarked upon by several of those who witnessed the operation, and during its removal numerous veins were noticeable upon the under side of the growth, which stood out in their distended condition and showed a characteristic bluish color.

By the time this step was completed, the patient's condition was as bad again, the pulse weak and the skin moist. The usual dressings were applied; no attempt being made to unite the edges of the wound and the patient was removed to his room where a hypodermoclysis was promptly given to which was added four ounces of whiskey. His condition gradually improved but within five hours he was dead. The manner in which he died led to the inference that his death was due to a cardiac embolism.

The pathological findings of the specimens removed as made by the pathologist of the hospital, Dr. James A. Kelly, showed that the growth was that of a simple nevus.

Appendicostomy as an aid to the Treatment of Malignant and Intractable Dysentery, by John L. Jelks, M.D., Memphis, Tenn.

In reference to this subject, the author stated that when amebic infection had become very chronic or had extended into all the parts of the colon beyond the use of local measures, and in some instances, of acute malignant cases, appendicostomy should be performed and irrigation practised through the appendiceal stump. The water is allowed to pass out through the rectum into a catch-basin and is not an unpleasant method of treatment. Dr. Jelks prefers the method suggested by Dr. James P. Tuttle, of New York City, who conceived the plan of allowing the appendix to remain undisturbed after anchorage, for a sufficient time (three or four days), to establish adhesions about the proximal end, before cutting away the distal portion and using the appendical stump-lumen through which to irrigate with the desired solutions.

Dr. Jelks practised this method and irrigated the colon with formalin-boric, copper-phenol-sulphonate, quinine and normal salt solutions with gratifying results. It was observed, however, that irrigations thus given did not effect a cure. Topical applications (per sigmoidoscope or rectoscope) were in all cases used in conjunction.

The method as used by Weir, and as advised by Tuttle, is practically free from danger, and the author believes is not more hazardous than appendicostomy and the after-effects are not at all unpleasant to the patient in the ways and degrees that a colostomy must be. He sees no great danger of hernia or wound infection if proper precautions are taken in dressing the same. By this method one may practise almost continuous irrigation of an inflamed colon and rectum with no special degree of pain or discomfort to the patient—the appendix being used as a nozzle, directing the solution into the colon.

He does not advise appendicostomy except in a small percentage of cases, mostly chronic ones, but in these, he insists that it is a most valuable aid to treatment and that the operation itself is practically free from danger, as is appendectomy when the appendix is not the seat of infection.

The author concludes his article by stating that in all cases requiring appendicostomy we should not permit the stump to close before the expiration of one year. He has been forced to reopen an appendical stump three months after closure and resume irrigations. This was accomplished in his office, but it may become a difficult matter to find the lumen of a closed appendix.

Primary Gonorrhea of the Rectum in the Male, by Alfred J. Zobel, M.D., San Francisco, Cal.

The writer stated that a review of the literature for the past five years showed very little to have been written on the subject of rectal gonorrhea, and the cases reported have been rectal gonorrhea in the female and for the most part secondary to an infection of the genital tract.

It was also stated that gonorrhea of the rectum in the male is almost always the result of sodomistic practises, and when so, can be considered of the primary type. The condition has been rather rare in this country but since the influx of foreigners from those countries where unnatural practises are common, more cases are now seen.

The cases reported by the writer were seen in the rectal clinic at the San Francisco Polyclinic and were in American born boys of 16, 18 and 20 years of age, respectively. They belonged to the tramp class and were of a rather low order of intelligence. They were ignorant of their true condition and came to the clinic with a self-made diagnosis of "piles." When made aware of the true nature of their trouble it had a markedly depressing effect upon them, which in one case, after a few weeks, developed into a condition resembling the neurasthenia which often accompanies a chronic posterior urethritis.

The symptoms complained of, briefly summarised, were: all complained of such soreness about anus and rectum that they did not care to stand; while walking was an effort and caused great pain. At the time of bowel movement they suffered such excruciating pain that they hesitated to pass their feces, and had become quite constipated. Two were annoyed by discharge from the anus, while one was unaware of its presence, although it was found on examination. In one, the discharge was streaked with blood, and bleeding was noticed at the time of defecation. One complained of an itching sensation about an inch up from the anal aperture, and had severe pain on the drawing in of the anal sphincters. Their appearance was feverish, worried and haggard, and they felt weak, ill and distressed.

It was impossible to make a digital or instrumental examination at the first visit on account of the severely acute pain caused thereby. Therefore, whenever there is the least suspicion of the possibility of a specific inflammation of the anus and rectum, the case should be treated as if it actually exists, and the ultimate diagnosis left to the future. When the acute symptoms have subsided under treatment, there can be seen excoriations and fissures about the anal orifice and in the canal, with marked redness and infiltration of the mucous membrane of the anus and rectum, together with the presence of a purulent secretion. Examination of this secretion shows the presence of the gonococcus.

The author believes that gonorrhea of the rectum in the male is a much more common condition than is suspected by the general profession. Many of the latter even do not know that such a condition could exist.

The treatment is directed towards keeping the parts clean; relieving the severe rectal symptoms; reducing the inflammatory exudates; keeping the fecal movement soft; healing the ulcerations and destroying the infective agent.

The author further brings out the important point, which he deems worthy of consideration, that there seem to be no reasons why complications, such as gonorrheal arthritis or an endocarditis could not arise. While so far as he is aware, no cases of an endocarditis or an arthritis resulting from rectal gonorrhea have been reported, yet it would be well for the internist to bear in mind that an examination of the rectum might furnish the clue in a baffling case, where the etiological factor is missing.

Operation for Anal Pruritus, by Thomas Charles Martin, M.D., of Washington, D. C.

The use of a solution of cocain and adrenalin secures local anesthesia and a dry visible field. Radiating incisions do not endanger the nutrition of the parts. Corrugation of the flaps may be effaced by traction of their margins. A skin-tag may be removed within an eliptic incision, which by suture may be given a linear form. Radiating wounds require no suture, coaptate automatically when the patient is in extension, and heal by first intention.

